# The Importance of Orthostatic Increase in Pulse Wave Velocity in the Diagnosis of Early Vascular Aging

**DOI:** 10.3390/jcm13195713

**Published:** 2024-09-25

**Authors:** Victor Dorogovtsev, Dmitry Yankevich, Andrey Martyushev-Poklad, Ilya Borisov, Andrey V. Grechko

**Affiliations:** Federal Research and Clinical Center of Intensive Care Medicine and Rehabilitology, 107031 Moscow, Russia; dyankevich@fnkcrr.ru (D.Y.); avmp2007@gmail.com (A.M.-P.); realzel@gmail.com (I.B.); noo@fnkcrr.ru (A.V.G.)

**Keywords:** preclinical orthostatic hemodynamic dysregulation, head-up tilt test, pulse wave velocity, vascular stiffness, vascular aging, orthostatic increase in vascular stiffness, arterial hypertension, risk-based prevention

## Abstract

**Background/Objectives**: Vascular aging can be assessed by arterial stiffness measured through pulse wave velocity (PWV). Increased PWV predicts arterial hypertension, cardiovascular events and all-cause mortality. Detection of early signs of vascular aging remains an unmet problem. To search for the most sensitive markers for the early increase in vascular stiffness in a healthy population. **Methods**: One-hundred and twenty healthy subjects were divided in three equal age groups: <30 years, 30–45 years and >45 years. Head-up tilt test (HUTT) protocol was applied, providing a standardized hydrostatic column height. PWV at the brachial–ankle artery site (baPWV) was measured using a multichannel sphygmomanometer ABI System 100 PWV in three positions: in the baseline horizontal (supine) position—baPWVb; during the head tilt-up with an individual angle of inclination—baPWVt; and when returning to supine. **Results**: The most sensitive marker of early stiffness increase in a healthy population is the relative orthostatic increase in baPWV, ΔbaPWV/baPWVb, where ΔbaPWV = baPWVt − baPWVb. The significance of differences in this parameter between the young and elderly groups reached *p* = 0.000075 and *p* = 0.000006, respectively. **Conclusions**: The proposed index ΔbaPWV/baPWVb can be considered as a promising sensitive early biomarker of vascular aging and as a potential effective indicator in cardiovascular prevention. A longitudinal cohort study is needed to confirm this assumption.

## 1. Introduction

Vascular stiffness is one of the most important independent risk factors for arterial hypertension (AH) and other cardiovascular diseases (CVD), and is also associated with increased all-cause mortality [1,2,3,4,5,6,7,8,9]. Progressive age-related structural changes in the vascular wall that increase stiffness include endothelial cell alterations, elastin fragmentation, collagen accumulation and medial vascular smooth muscle cell remodeling [10,11,12,13,14]. An important factor that affects arterial stiffening in muscular or mixed arteries is an increase in intima–media thickness [15,16,17]. The mentioned age-related changes in the structure of the vascular wall point at the relevance of vascular stiffness for the evaluation of biological age [18,19,20,21,22].

The most common method for assessing vascular stiffness is the measurement of carotid–femoral pulse wave velocity (cfPWV) [23,24,25,26]. This method allows the determination of the type of vascular aging: normal, supernormal or early (accelerated) [27,28,29,30]. Importantly, vascular stiffening can significantly accelerate in various age-related conditions: AH, Diabetes mellitus, metabolic syndrome, obesity, chronic systemic inflammation, chronic kidney disease, etc. [31,32,33,34]. Thus, early detection of vascular stiffening and accelerated vascular aging is very relevant for prevention. The current criteria of normal age-specific cfPWV values have a broad range, which makes it difficult to detect early stages of accelerated vascular aging in young individuals without distinct health conditions [18,35,36,37]. To solve this problem, it is necessary to identify the most informative early indicators of vascular aging able to identify the initial increase in vascular stiffness in a healthy population. It is important to keep in mind that age-specific PWV reference values are developed for the horizontal position of the body (full-functional resting state).

Upon orthostasis, the cardiovascular system experiences natural stress, which tests the strength of all adaptation mechanisms. Their inadequacy manifests, among other things, in the form of orthostatic hypo- and hypertension. The head-up-tilt test (HUTT) is a way to reproduce these conditions of natural stress that occurs when shifting from a horizontal to vertical position. The test was initially proposed for the diagnosis of syncope; its standard procedure, opportunities and limitations are described in detail [38].

We consider the study of preclinical asymptomatic orthostatic hemodynamic dysregulations in a healthy population to be a promising approach to detect early signs of vascular aging. We have developed a specific HUTT protocol to investigate preclinical orthostatic abnormalities in healthy subjects [39]. It can predict AH, early vascular remodeling and increased vascular stiffness [40,41,42]. During orthostasis and HUTT, vascular stiffness increases due to stimulation of the sympathetic baroreflex and an adaptive neurohormonal shift with activation of pressor systems that increase peripheral vascular resistance [43,44,45]. This process is necessary to maintain the stability of organ blood flow during changes in body position.

We realized that cfPWV mainly reflects the state of the aorta, a large elastic artery. For the study of orthostatic changes, we preferred to use brachial–ankle pulse wave velocity (baPWV) because it reflects the functional state of both the large elastic and medium and small muscular arteries, which are actively involved in adaptive processes. This is the major site of remodeling occurring in the muscular layer in arterial hypertension [46].

In our previous work, we studied the phenotypes of preclinical orthostatic abnormalities, age-related characteristics of baPWV and its orthostatic changes in different age groups of healthy subjects. The article was published in MDPI Diagnostics [42]. In this work, we have shown an age-dependent increase in baPVWV. The present work focuses on an in-depth analysis of orthostatic changes in vascular stiffness detected in the subjects of our previous study. We considered that even asymptomatic orthostatic abnormalities other than orthostatic normotension are predictors of AH and are associated with early remodeling of the vascular wall and an increase in its stiffness. Therefore, the aim of this work was to identify the most sensitive markers for the early increase in vascular stiffness in a healthy population.

## 2. Materials and Methods

### 2.1. Participants

Inclusion criteria: adults without an exacerbation of an existing illness, non-smokers and not taking coffee or alcohol 24 h prior to experiment. Individuals with an established diagnosis of chronic disease were included if their clinical and laboratory parameters corresponded to the age norm during annual health check-ups.

Exclusion criteria: cardiac arrhythmias, acute heart disease, history of CVD, peripheral arterial blood flow disorders, history of orthostatic intolerance, peripheral edema, signs of thrombophlebitis or complicated varicose veins and subjects taking betablockers or diuretics with blood pressure above 140/90 mmHg with body mass index over 30 kg/m^2^.

The uniqueness of this study lies in the meticulous selection of healthy individuals across different age groups who had no history of orthostatic intolerance episodes or cardiovascular events. This selection was based on a thorough preventive examination that included clinical, biochemical, electrophysiological, and radiological assessments. Participants were chosen by controlling for several confounding factors: age, sex, body mass index, smoking status, systolic blood pressure, serum cholesterol levels, and diabetes. All individuals with values outside the normal range or those who smoked were excluded from the study. Due to the relatively small sample size within age groups, we did not analyze the impact of gender as a confounding factor on orthostatic changes. Measurements were conducted two hours after a light breakfast, between 8:00 and 11:30 a.m., in a laboratory environment maintained at an ambient temperature of 24 to 25 °C, with 50–55% humidity and minimal noise levels.

### 2.2. Head-Up Tilt Test (HUTT) Procedure

The HUTT was conducted using an electrically operated tilt table following the Luanda protocol, which comprised three phases: supine position for 10 min, HUTT position (tilt) for 10 min, and return to supine position for 10 min. This protocol involved setting an individualized tilt angle to standardize the hydrostatic column height for all participants, regardless of their actual height [39].

### 2.3. Hemodynamic Measurements

Two brachial and ankle pulse wave velocity (baPWV) measurements from the 6th to 10th minute at each of the three positions were performed using the multichannel volumetric sphygmography method on an ABI System 100 PWV (BOSO, Germany). In each of the three positions, baPWV was measured twice and averaged. In addition to measuring BP and baPWV, this device performed a computerized calculation of carotid–femoral pulse wave velocity (cfPWV), and the accuracy of the calculation of these parameters was validated [47,48].

### 2.4. Statistical Analysis

Statistical analysis was carried out using STATISTICA v.10 software (StatSoft^®^, Tulsa, OK, USA). Nominal data were presented as absolute values. For quantitative data with a normal distribution, the results were organized into variation series, with the arithmetic mean (M) and standard deviations (SD) calculated. In cases where the data did not follow a normal distribution, quantitative values were expressed as the median and quartiles (25–75% interquartile range). The Kolmogorov–Smirnov test was used to determine the nature of the data distribution. For comparing mean values in normally distributed quantitative data, the student’s *t*-test was applied. When comparing two independent groups where the data did not follow a normal distribution, the Mann–Whitney U-test was used. Kruskal–Wallis’s test was utilized for comparing multiple samples of quantitative data with non-normal distributions. To evaluate the statistical significance of differences in quantitative characteristics between two dependent samples, the Wilcoxon W test was employed. A *p*-value of less than 0.05 was considered statistically significant.

Linear regression was used to analyze the relationship between the dependent and independent variables. The results of the analysis were presented in the form of regression coefficients, determination coefficients and *p*-values.

## 3. Results

The key findings of the present study, the orthostatic changes in baPWV during HUTT in different age groups, are presented in Table 1.

The following differences in baseline data were found in the analysis of the averages of the three age groups: lower HR and DBP values in group 1 compared with group 2 (*p* = 0.004) with no significant difference in SBP. During HUTT, there was an increase in HR and DBP in all groups, with little change in SBP, and the differences between the groups were not significant (Table 1).

The baPWVb values in the initial horizontal position were statistically significantly different across the groups. The minimum baPWVb values (lowest vascular stiffness) were observed in group 1, and the maximum values—in the older age group 3. The differences between all the groups were statistically significant (Table 1).

When analyzing orthostatic changes in vascular stiffness, we found a statistically significant increase in baPWVt compared to baPWVb in all three groups (*p* = 0.001) [42]. An intergroup analysis of baPWVt during HUTT revealed that under orthostasis, differences in arterial stiffness between groups largely leveled out, except for significant differences in baPWVt between groups 1 and 3.

The increase in baPWV during HUTT (ΔbaPWV = baPWVt − baPWVb) was maximal in group 1, was significantly lower in group 2, and ΔbaPWV was minimal in group 3. The differences in ΔbaPWV between groups 2 and 3 were insignificant. We paid attention to the fact that with age, baPWVb (vascular stiffness at rest) tends to increase, and ΔbaPWV (stiffness increase during functional orthostatic load) decreases.

The most striking differences between the groups were found in the relative increase in vascular stiffness during orthostasis (ΔbaPWV/baPWVb) (Table 1).

**Figure 1:**
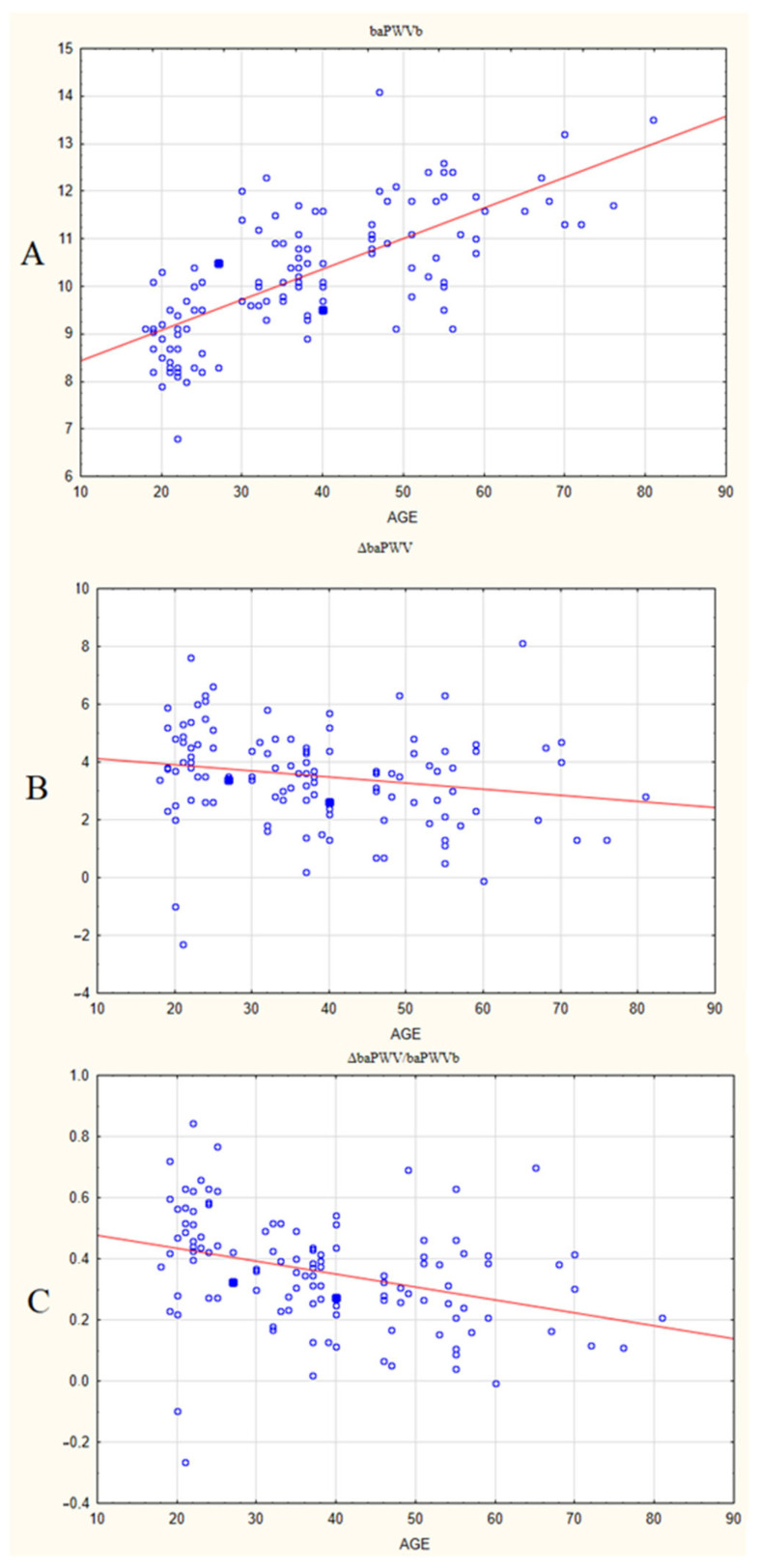
Linear regression graphs between age (independent variable) and different indices of vascular stiffness in healthy individuals: basal PWV, baPWVb (**A**), increase in baPWB after tilt, ΔbaPWV (**B**), and relative increase in baPWV after tilt, ΔbaPWV/baPWVb (**C**). Legend: Horizontal axis: age, years. Vertical axis: baPWVb, m/s (**A**), ΔbaPWV, m/s (**B**) and ΔbaPWV/baPWVb (**C**). Red line—regression line in linear model of regression.

Graphs showing linear regression between age (independent variable) and various indices of vascular stiffness during orthostasis are presented in Figure 1.

The basal baPWV shows a relationship with age, with a positive regression coefficient beta = 0.712. This suggests that with aging, vascular stiffness at rest (in the horizontal position) increases. This connection is strong and statistically significant, with *p*-value < 0.0001. The determination coefficient (R^2^) for this pair is 0.507, which means that 50.7% of the baPWV variation at rest can be attributed to aging. The corrected determination coefficient (R^2^ corrected) is 0.50289; thus, a major part of baPWV variation can be explained by aging.

For the increase in PWV after tilt (ΔbaPWV), the relationship with age is different; it is weak and negative: regression coefficient beta = −0.191, (*p*-value is <0.05). For this pair, R^2^ = 0.0367 and R^2^ corrected is 0.0285. Therefore, age is not a significant contributor to variation in ΔbaPWV.

Finally, the relative change in PWV after tilt (ΔbaPWV/baPWVb) shows a moderate negative connection with age, with beta = −0.348 (*p*-value < 0.0001), R^2^ = 0.1215 and corrected R^2^ = 0.114. Therefore, age is a more significant contributor to variation in ΔbaPWV/baPWVb than in ΔbaPWV; however, there are other more important factors.

## 4. Discussion

In the present study, we identify three indices of vascular stiffness which are measured in healthy adults of different age groups during orthostasis (head-up tilt). Below, we will discuss how they might reflect different aspects of age-related cardiovascular adaptation to this type of natural stress.

From the point of view of thermodynamics, life is characterized by a stable pronounced non-equilibrium state, significantly distant from entropy. The human organism is a complex, stable, non-equilibrium system in relation to the environment. It is in a constant state of flux, striving to maintain its non-equilibrium and stability, and expending a considerable amount of energy to do so.

Biological age is determined by the degree of viability of the organism, i.e., the ability of the body’s functional systems to effectively adapt to the constantly changing environment. At the same time, biological age is largely determined by the state of the vascular system, i.e., vascular age.

The measure of vascular age is the adaptive capacity of the vascular system (hemodynamic reserve) to maintain and fine-tune adequate organ blood flows in response to changes in metabolic needs and in hydrostatic pressure (the latter is mainly associated with changes in the position of the body in space). Throughout the human life span, a person’s activity in the environment determines their need for fine and proper regulation of the cardiovascular system to neutralize this increasing hydrostatic pressure to maintain organ blood flow at a level appropriate to their needs. The inadequacy of orthostatic regulation of hemodynamics leads to an early increase in vascular stiffness, which determines the need to detect its early manifestations in a healthy population.

In our work, we focus on the understudied initial preclinical stage of this process in a healthy population, even before the onset of structural pathological changes.

Our data indicate that vascular stiffness in the HUT position (baPWVt) includes two components, which together are designed to ensure adequate adaptation of the vasculature (hemodynamics) to increased hydrostatic pressure. The first component, baPWVb—pulse wave propagation velocity in the horizontal position (under functional rest)—largely depends on the current state of the vascular wall structure, and, to a much lesser extent, on the neurohormonal background. This index increases significantly with age, which is a sign of vascular aging. Our study supports that calendar age by itself is a major contributor to vascular age. The second component, ΔbaPWV, appears under increased hydrostatic load and reflects the capacity for dynamic adaptation of blood flow and the cardiovascular system in general, since it results from sympathetic baroreflex activation and a significant neurohormonal shift with the activation of pressor systems [43,44,45,49,50,51,52,53,54]. Therefore, this component can be defined as a functional reserve of the orthostatic regulation of hemodynamics; this index determines the fine-tuning of orthostatic regulation, the main purpose of which is to adjust an adaptive response to the degree of increase in hydrostatic pressure.

As shown in our study, this functional reserve (the value of ΔbaPWV) has a broad variation, and age is not a significant contributor to this dispersion. Therefore, there are other factors that affect dynamic adaptation, and very probably, they are multiple and reversible. Some potential mechanisms that affect the functional reserve are those that emerge long before a clinical disease. They are related with universal mechanisms of accelerated aging:

- chronic distress (including oxidative stress) may promote an age-related decline in functional reserves due to vegetative imbalances and sympathetic hyperactivity, which increases with age [55,56].
- chronic low-grade systemic inflammation and insulin resistance both affect endothelial function and vascular remodeling.

This means that not only do orthostatic changes occur in the neurohormonal background, but also its age-related changes may contribute to increased vascular stiffness. In our opinion, the value of ΔbaPWV can only characterize the state of the vasculature in the context of baseline vascular stiffness (baPWVb). Therefore, we propose that an index that takes account of both ‘static’ age-related changes and dynamic adaptation should be the most relevant predictor of age- and lifestyle-related cardiovascular disadaptation.

We believe that orthostatic circulatory dysregulation is more important in increasing vascular stiffness. This is confirmed by higher indices in subjects with clinical orthostatic hypotension compared to the same age group with orthostatic normotension [57,58,59]. In this regard, the remodeling of the vascular wall with an increase in its stiffness can be considered as a compensation aimed at increasing the orthostatic stability of blood flow. Such remodeling is reflected in the value of baPWVb, which tends to increase with age, with significant intergroup differences (*p* < 0.001).

Thus, age-dependent changes in baPWVb reflect the aging of the vascular system as an inadequate compensatory response to an age-dependent decrease in the adaptive capabilities of the organism to maintain the sufficient perfusion of organs in any body position. These adaptive capabilities are partly reflected in the ability to dynamically increase stiffness under orthostatic loading (ΔbaPWV), which tends to decrease with age. The objectives of our study did not include the analysis of the cause–effect relations of these two components, but our data allow us to continue the search for the most informative indicator of early vascular aging and to find an integrative indicator combining structural and functional components.

According to our data, the most sensitive predictor of early aging and age-dependent vascular changes may be our proposed index of relative increase in baPWV during HUT (ΔbaPWV/baPWVb). It considers the structural (baPWVb) and the functional (ΔbaPWV) components of adaptation that change differently with age. We expect that it will most fully measure the hemodynamic reserve of the vascular system, which decreases due to age-related changes, i.e., with increasing vascular age.

We can assume that the ΔbaPWV/baPWVb index will be very informative in the personalized analysis of vascular aging and vascular system state, both natural and in response to any treatment, lifestyle modification or other measures designed to predict and prevent AH and other CVDs.

## 5. Conclusions

In this paper, we revealed a connection between aging and cardiovascular adaptation to a major stressful factor of the environment: orthostasis.

Our results imply that adaptation to orthostasis involves two aspects: dynamic adaptation, which can be detected in the tilt-up position, and static adaptation, which can be detected as an increase in baseline vascular stiffness.

We proposed an index (relative increase in vascular stiffness during tilt test, ΔbaPWV/baPWVb) that has a potential to reflect both the age-related and lifestyle-related disadaptation of the cardiovascular system to orthostasis.

## 6. Limitations

Our study involved individuals without clinical signs of cardiovascular or metabolic diseases. However, they might have had certain preclinical conditions which could affect vascular aging, such as chronic distress, oxidative stress, chronic low-grade inflammation or insulin resistance. Due to our small sample size, we also could not reveal how the studied indices are affected by gender.

## 7. Future Directions

There are two major aspects of research that we consider worth performing with special focus on early vascular aging:

(1) The impact and contribution of lifestyle-related preclinical conditions underlying accelerated aging (such as chronic distress, chronic low-grade inflammation and insulin resistance) on the ‘static’ and ‘dynamic’ components of cardiovascular disadaptation to orthostasis presented by the baPWVb and ΔbaPWV/baPWVb indices.
(2) It is expedient to perform a long-term (longitudinal, prospective) study to evaluate how the newly proposed indicator of vascular aging will change with time, and how the ΔbaPWV/baPWVb index will correlate with preclinical orthostatic abnormalities and endothelial function, as well as with chronic inflammation and oxidative stress, in a healthy population.

## Figures and Tables

**Table 1:** Comparative analysis of orthostatic changes in heart rate (HR), systolic blood pressure (SBP) and baPWV index in different age groups of healthy subjects.

Parameters	Group 1	Group 2	Group 3	Inter-Group Differences			KW
Average Age	*n* = 40	*n* = 40	*n* = 40				
[Age Range], Years	22 [20; 24]	37 [33; 38]	55 [49; 59]	*p*_1–2_	*p*_1–3_	*p*_2–3_	
HRb b/min	62 [57; 67.5]	68 [62.5; 77.5]	64.5 [58; 72]	0.004	0.39	0.29	10.01 *p* = 0.007
HRt b/min	74 [68.5; 78]	73 [69.5; 85]	67.5 [62.5; 75]	0.059	0.063	0.015	8.97 *p* = 0.011
SBPb mmHg	120.3 [109.7; 126]	125.1 [119; 130.6]	125.6 [115; 136.6]	0.05	0.1	0.058	6.9 *p* = 0.03
SBPt mmHg	123.1 [108.5; 128.5]	126.3 [113.2; 134.5]	120.2 [112.8; 131.1]	0.25	0.061	0.059	3.0 *p* = 0.223
DBPb mmHg	72.7 [66.2; 75.7]	81.5 [73.6; 86.8]	82.5 [73; 89.8]	0.001	0.001	0.054	25.75 *p* < 0.0001
DBPt mmHg	79.9 [74; 82.5]	85.9 [79.4; 90.7]	84.3 [77.5; 90.8]	0.003	0.051	0.09	11.45 *p* = 0.003
baPWVb, m/s	8.9 [8.3; 9.5]	10.1 [9.7; 11.0]	11.3 [10.7; 11.9]	0.001	0.001	0.001	66.7 *p* < 0.0001
baPWVt, m/s	12.9 [11.9; 14.2]	13.8 [13.0; 14.5]	14.3 [13.3; 15.4]	0.16	0.001	0.26	13.3 *p* = 0.0013
ΔbaPWV, m/s	4.0 [3.45; 5.15]	3.55 [2.7; 4.35]	3.05 [1.95; 4.35]	0.023	0.007	0.34	8.83 *p* = 0.012
ΔbaPWV/ baPWVb	0.45 [0.41; 0.58]	0.35 [0.25; 0.42]	0.27 [0.16; 0.39]	0.000075	0.000006	0.073	26.1 *p* < 0.0001

Note: Keys to abbreviations: SBPb—baseline systolic blood pressure; SBPt—systolic blood pressure during HUT; DBPb—baseline diastolic blood pressure; DBPt—diastolic blood pressure during HUT; HRb—baseline heart rate; HRt—heart rate during HUT; baPWVb—baseline pulse wave velocity in supine position; baPWVt—pulse wave velocity during HUT; ΔbaPWV—difference between baPWVt and baPWVb; ΔbaPWV/baPWVb—relative increase in baPWV during HUT (ratio of ΔbaPWV to baPWVb). For parameters that were not distributed normally, the data are presented as median [range]; KW—Kruskal–Wallis’s test.

## Data Availability

Data are contained within the article.
